# The deaf mouse mutant whirler suggests a role for whirlin in actin filament dynamics and stereocilia development

**DOI:** 10.1002/cm.20199

**Published:** 2007-02-26

**Authors:** Mette M Mogensen, Agnieszka Rzadzinska, Karen P Steel

**Affiliations:** 1 School of Biological Sciences, University of East AngliaNorwich, United Kingdom; 2 Wellcome Trust Sanger Institute, Genome CampusHinxton, Cambridge, United Kingdom

**Keywords:** stereocilia, actin filaments, deafness, whirlin, hair cells, inner ear, organ of Corti, cochlea

## Abstract

Stereocilia, finger-like projections forming the hair bundle on the apical surface of sensory hair cells in the cochlea, are responsible for mechanosensation and ultimately the perception of sound. The actin cytoskeleton of the stereocilia contains hundreds of tightly cross-linked parallel actin filaments in a paracrystalline array and it is vital for their function. Although several genes have been identified and associated with stereocilia development, the molecular mechanisms responsible for stereocilia growth, maintenance and organisation of the hair bundle have not been fully resolved. Here we provide further characterisation of the stereocilia of the whirler mouse mutant. We found that a lack of whirlin protein in whirler mutants results in short stereocilia with larger diameters without a corresponding increase in the number of actin filaments in inner hair cells. However, a decrease in the actin filament packing density was evident in the whirler mutant. The electron-density at the tip of each stereocilium was markedly patchy and irregular in the whirler mutants compared with a uniform band in controls. The outer hair cell stereocilia of the whirler homozygote also showed an increase in diameter and variable heights within bundles. The number of outer hair cell stereocilia was significantly reduced and the centre-to-centre spacing between the stereocilia was greater than in the wildtype. Our findings suggest that whirlin plays an important role in actin filament packing and dynamics during postnatal stereocilium elongation.

## INTRODUCTION

Highly specialised epithelial cells, the sensory hair cells in the cochlea, are responsible for mechanoelectrical transduction and ultimately the perception of sound. The apical surface of each hair cell contains a staircase-like bundle of mechanosensory stereocilia [Slepecky and Ogata, 1996]. Sound induced mechanical vibrations that cause bundle deflection towards the taller row are thought to stretch interstereocilial links, opening transduction channels and allowing influx of cations that depolarise the hair cell and generate a receptor potential [Hudspeth, 1985]. Stereocilia stiffness and function depend on the several hundreds of uniformly polarised and tightly cross-linked actin filaments which make up the paracrystalline array of their core. The actin filaments are oriented with the barbed-ends (plus and fast growing ends) at the apex where they are embedded in the tip-complex [DeRosier and Tilney, 2000]. Recent studies on stereocilia dynamics have revealed that the stereocilia actin cytoskeleton shows continuous turnover with actin filament assembly occurring at the stereocilium tip and its disassembly at the base. Total actin filament renewal within the stereocilia thus occurs by a treadmilling mechanism with turnover rates proportional to the stereocilia lengths [Rzadzinska et al., 2004]. Murine stereocilia start to develop from dense apical plaques by embryonic day 14.5 [Nishida et al., 1998; Denman-Johnson and Forge, 1999]. The initial bundle of microvilli-like stereocilia of uniform length gradually differentiates so that those stereocilia which are in close proximity to the kinocilium (non-motile primary cilium) become longer and a staircase-like organisation of the hair bundle becomes apparent. In mammals the excess stereocilia at the front of the hair bundle are reabsorbed while the remaining stereocilia continue to grow simultaneously in length and width until they reach their predetermined size [Kaltenbach et al., 1994]. The fully formed stereocilium tapers at the base forming an ankle and a few actin filaments continue into the dense actin network of the cuticular plate within the top of the hair cell where they form an anchoring rootlet.

The molecular bases for stereocilia development, organisation and maintenance are not fully understood. Several proteins implicated in hearing loss in humans and mice have been associated with malformations of the stereocilia bundles. Interestingly, mutations in three different genes, *Espn, Myo15a* and *Whrn* (encoding espin, myosin XVa and whirlin respectively) produce similar phenotypes with stereocilia of affected mice being significantly shorter than their wildtype litter-mates [Sjostrom and Anniko, 1992; Probst et al., 1998; Zheng et al., 2000; Mburu et al., 2003]. *Espn* encodes one of the actin filament cross-linkers in stereocilia, which is uniformly distributed within the actin array [Rzadzinska et al., 2005]. Mutations in the espin gene affect stereocilia stability, which results in their progressive postnatal shortening in the *jerker* mouse until they disappear completely from the surface of adult hair cells [Sjostrom and Anniko, 1992; Zheng et al., 2000]. Myosin XVa, an unconventional myosin, is present at the stereocilia tips from the initial stages of stereocilia development and throughout adulthood at a level proportional to the length of the stereocilium [Rzadzinska et al., 2004]. When the *Myo15a* gene is mutated, the stereocilia are short and lack a tip-complex [Probst et al., 1998; Rzadzinska et al., 2004].

The whirlin gene encodes a novel PDZ protein, which transiently localises to stereocilia tips during their development [Mburu et al., 2003, 2006; Belyantseva et al., 2005; Delprat et al., 2005; Kikkawa et al., 2005]. Mutations in the human whirlin gene are responsible for autosomal recessive deafness at locus DFNB31 in humans and for the deafness phenotype in the whirler mouse [Mburu et al., 2003]. Recently mutations in *WHRN* have been found also in cases of Usher syndrome type 2D in humans [Ebermann et al., in press]. Previous scanning electron microscope studies have revealed that in adult whirler homozygotes the stereocilia of the inner hair cells are significantly shorter than in controls due to a progressive decrease in length during postnatal development. In contrast, outer hair cells of whirler mutants showed irregularities in the shape of the hair bundles with some shorter stereocilia present [Holme et al., 2002]. Recent *in vitro* studies indicate possible interactions between myosin XVa and whirlin during stereocilia development and suggest that myosin XVa may be necessary for whirlin localisation at stereocilia tips [Belyantseva et al., 2005].

Here we have exploited high resolution transmission and scanning electron microscopy to further characterise the stereocilia bundles of adult whirler mice. We found that the short stereocilia of the whirler mutant were wider than the controls but that this increase in width was not accompanied by a significant increase in the number of actin filaments. We also found that the short whirler stereocilia contained patchy electron densities at their tips. The specific changes observed within the hair bundles of the whirler mutant varied between the inner and outer hair cells. Progressive reduction in the length of whirler stereocilia and changes in their width together with the developmental expression pattern for whirlin support the hypothesis that whirlin plays crucial roles in actin filament dynamics and stereocilia development. Furthermore, our results suggest that whirlin's role in stereocilia development is at least in part distinct from that of myosin XVa.

## MATERIALS AND METHODS

### Mice

Whirler mutants were originally obtained from Dr E Rinchik, Oak Ridge National Research Laboratory [Rinchik et al., 1991] and were maintained on their original (undefined) genetic background. All experiments were carried out in compliance with UK Home Office regulations.

### Electron Microscopy

Cochleas from two *wi*/*wi* mice, one +/*wi* mouse and one +/+ mouse at P20 were processed for transmission electron microscopy (TEM) analyses. Freshly dissected cochleas were pierced at the apex and fixed for 2 h in 2.5% glutaraldehyde followed by 1% osmium tetroxide each in 0.1 M sodium cacodylate with 2 mM calcium chloride. They were then dehydrated in ethanol, en bloc stained with uranyl acetate (1% solution of uranyl acetate in 100% ethanol) and embedded in araldite resin. Ultrathin sections (50–70 nm) were collected from the middle turn of the cochlea, between 50–60% of the cochlea length. The sections were all stained in uranyl acetate and lead citrate and analysed in the TEM. The stereocilia diameter, number of actin filaments and nearest-neighbour distances between them were analysed in good orthogonal profiles of the inner and outer hair cell stereocilia from the most lateral row. Longitudinal sections were obtained through the middle plane of inner hair cell stereocilia. Two whirler homozygotes and two controls (one wildtype and one heterozygote) were used for these analyses. Analyses of inter-stereocilia distances within and between rows were based on measurements of the centre-to-centre distance between adjacent stereocilia in hair bundles containing good cross-sectional profiles as above. To analyse packing density of the actin filaments the number of filaments was counted within arbitrarily placed 8610 nm^2^ squares and the distances between the closest actin filaments were measured. In addition, we estimated the mean predicted distances between actin filaments from the equation for nearest-neighbour distances for really random points: *S* = *Nd*^2^ (*S*, cross-sectional area of an average actin bundle within stereocilium; *N*, the number of actin filaments; *d*, distances between nearest filaments) based on the measured cross-sectional area of the average actin bundle and the counted average number of actin filaments [Hsieh et al., 1994]. Post acquisition image analyses were performed using Adobe Photoshop CS2 and NIH Image software. *t*-tests were performed using Excel software.

For scanning electron microscopy (SEM), freshly dissected tissues from P20 mice (*wi*/*wi n* = 5; +/*wi n* = 5) were fixed for 3 h with 2.5% glutaraldehyde in 0.1 M sodium cacodylate buffer pH 7.4 with 2 mM CaCl2, processed using an OTOTO method [Hunter-Duvar, 1978], dehydrated in acetone, critical point dried, sputter coated with gold and viewed on a Hitachi 4800 FE Scanning Electron Microscope operated at 5 kV. Post acquisition image analyses were performed using Photoshop CS2 and NIH Image software. The length of the stereocilia from the tallest row was measured using NIH Image software and used for quantitative analyses. A minimum of 10 inner and 10 outer hair cells were collected from the middle turn of the cochlea (50–60% of the cochlear length) from three different animals of each genotype. All images were taken at the same distance from the detector and the samples were mounted at a comparable angle to minimize measurement errors. *T*-tests were performed using Excel software.

## RESULTS

This study showed no apparent ultrastructural differences between the stereocilia of heterozygote and wildtype mice. No abnormalities were evident in the cell bodies or the cuticular plates of the whirler homozygous hair cells compared with their control littermates at P20, as assessed by TEM (data not shown). However, distinct abnormalities were observed in the dimensions and organisation of the stereocilia of both the inner and outer hair cells of whirler homozygous mice.

### Stereocilia of Whirler Inner Hair Cells are Short and Wide

**Fig. 1:**
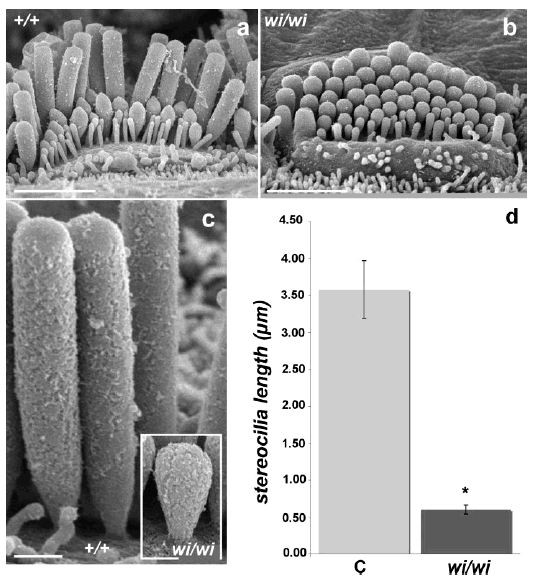
Stereocilia of whirler homozygote inner hair cells are short but a staircase-like organisation is still evident. SEM images showing inner hair cell stereocilia in the middle turn of the cochlea of a heterozygote control (**a**) and a homozygous whirler mutant (**b**) at P20. Note the appearance of a staircase-like organisation of short *wi*/*wi* stereocilia and increased number of stereocilia rows. **c:** SEM images of the tallest row of inner hair cell stereocilia in +/*wi* (main image) and *wi*/*wi* (inset) at the same magnification. **d:** Quantitative analysis based on SEM measurements revealed that the tallest of *wi*/*wi* inner hair cell stereocilia (*n* = 68) are considerably shorter than the tallest stereocilia of +/*wi* (*n* = 92) in the same region of the cochlea (50–60% of cochlear length). Standard deviation bars are marked. ^*^ represents a significant difference, *P* < 0.001. Scale bars: a and b = 2 μm; c = 200 nm.

**Fig. 2:**
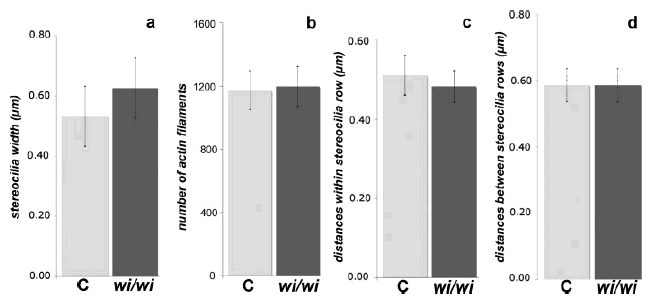
Inner hair cell stereocilia of whirler homozygotes are wider but show no significant increase in the number of actin filaments. Quantitative analysis based on TEM images showed that the tallest stereocilia of the *wi*/*wi* inner hair cells are significantly wider than those of the control (*P* < 0.05) (**a**) but that the number of actin filaments in their core did not increase significantly (**b**). The centre-to-centre distances between stereocilia within the same row were reduced in *wi*/*wi* hair bundles (*P* < 0.05) (**c**) but no change was observed in distances between stereocilia rows (**d**). Standard deviation bars are marked.

The stereocilia of inner hair cell heterozygote and wildtype mice were arranged in distinct rows of decreasing height. The tallest stereocilia with rounded tips formed the most lateral row. Stereocilia of the middle row were much shorter and showed characteristic pointed tips, while the morphology of the small stereocilia of the shortest row was similar to that of microvilli (Fig. 1a). In contrast, the tallest stereocilia of the adult whirler homozygote inner hair cells were significantly shorter (0.60 ± 0.06 μm, *n* = 68 stereocilia) than those of the controls (3.58 ± 0.39 μm, *n* = 92 stereocilia, *P* < 0.001) (Fig. 1d; measurements based on SEM images) and their tips were uniformly rounded (Figs. 1b–1c). Although not as well defined as in the control hair bundles, some degree of staircase-like organisation of the stereocilia was evident in the whirler inner hair cells (Fig. 1b). Instead of three distinct rows of stereocilia as found in wildtype mice, the whirler homozygotes had 4–5 rows of thick stereocilia of almost identical morphology (Fig. 1b). Detailed TEM analysis revealed that the tallest whirler stereocilia were significantly wider (0.63 ± 0.04 μm, *n* = 57, *P* < 0.001) than the tallest control stereocilia (0.53 ± 0.05 μm, *n* = 57) (Fig. 2a). The centre-to-centre distance between adjacent stereocilia within a row was shorter in the whirler mutant than in the control (+/+: 0.51 ± 0.05 μm, *n* = 52; *wi*/*wi*: 0.48 ± 0.04 μm, *n* = 52; *P* < 0.05), while no significant difference was observed in the distance between stereocilia rows (+/+: 0.59 ± 0.07 μm, *n* = 52; *wi*/*wi*: 0.59 ± 0.06 μm, *n* = 52; *P* = 0.96) (Figs. 2c and 2d). However, these differences in stereocilia width and position did not correspond to changes in the number of stereocilia within the tallest row (+/+: 15.03 ± 1.17 *n* = 28; *wi*/*wi*: 14.54 ± 1.23 *n* = 28, *P* = 0.1).

**Fig. 3:**
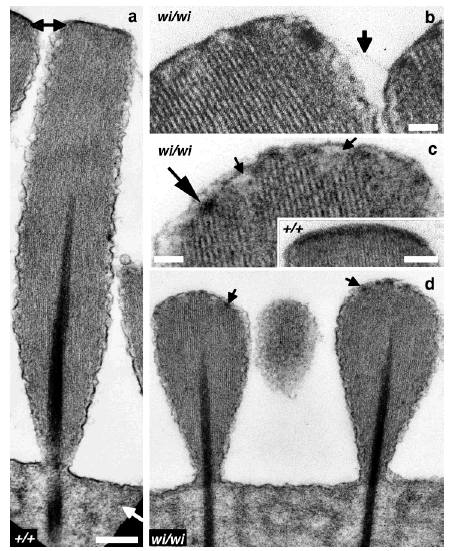
The electron density of the tips of whirler homozygote stereocilia has a patchy appearance. TEM images of longitudinal profiles through wildtype (+/+), and whirler homozygote (*wi*/*wi*) inner hair cell stereocilia at P20. (**a**) Parallel and tightly packed actin filaments form the core of the +/+ stereocilium and a few packed actin filaments form the rootlet passing through the ankle into the cuticular plate within the top of the hair cell (white arrow). At the tip of the stereocilia the continuous band of electron dense material (representing the tip-complex) is clearly visible (black double arrow). The presence of a continuous tip-complex in +/+ stereocilia tips is further highlighted in the inset in c. (**b**) A tip link (black arrow) connecting two *wi*/*wi* stereocilia is present and indistinguishable from those of the control. **c:** The electron density at the tip of a whirler homozygote stereocilium shows a patchy appearance (large arrow) and some areas close to the plasma membrane indicate regions where the actin filaments are shorter (small arrows). These actin filament-free areas appear to coincide with a lack of electron dense material at the tip. This is in contrast to the continuous electron density along the entire tip of a wildtype stereocilium (inset). (**d**) The actin core and the rootlet of *wi*/*wi* stereocilia show a normal ultrastructure but a patchy tip density is evident at the stereocilia tips (arrows). Scale bars: a = 0.25 μm and d at same magnification; b,c = 50 nm; c inset 100 nm.

**Fig. 4:**
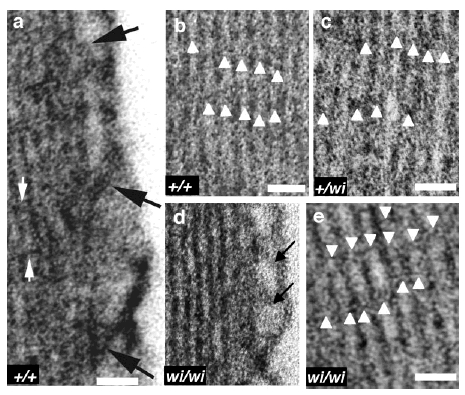
Actin filament cross-links in mouse stereocilia are aligned in register. TEM images of longitudinal profiles through +/+, +/*wi* and *wi*/*wi* inner hair cell stereocilia at P20 showing actin filament cross-links and arrangements. Regular links (black arrows) connect the stereocilium actin core to the plasma membrane in +/+ (**a**) and *wi*/*wi* (**d**). High magnification images showing part of the actin cores from +/+ (**b**), +/*wi* (**c**) and *wi*/*wi* (**e**) stereocilia with actin cross-links (highlighted by white arrowheads) showing transverse periodicity suggesting that the filaments are aligned in register. Scale bars: a = 40 nm and d at same magnification; b, c and e same magnification = 35 nm.

**Fig. 5:**
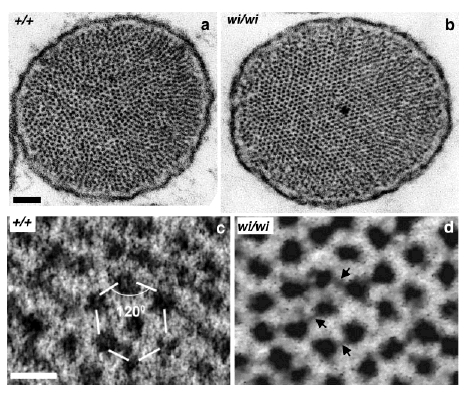
Actin filaments are organised in a hexagonal pattern and whirler actin bundles show reduced packing density. TEM images of cross-sectional profiles through +/+ (**a**,**c**) and *wi*/*wi* (**b**,**d**) inner hair cell stereocilia showing greater diameter in the whirler homozygote than in the wildtype (compare a and b). Note the distinct hexagonal arrangement of the actin filaments in both control (c) (highlighted with white lines) and whirler homozygote (d). (d) High magnification image of the actin filaments showing some of the cross-links (arrows) in a whirler homozygote. Scale bars: a and b = 60 nm; c and d = 20 nm.

In order to characterise the ultrastructure of the actin filament bundle of the whirler stereocilia, longitudinal and cross-sectional profiles of stereocilia were exam-ined to study the actin filament cross-links, the membrane-to-actin links and the number of actin filaments within their core. Wildtype, heterozygote and homozygote whirler stereocilia all contained an array of continuous parallel actin filaments extending from the apex to the base of each stereocilium with relatively few filaments forming the rootlet that reaches down into the cuticular plate (Figs. 3a and 3d). The actin filaments of the whirler stereocilia did not show any apparent breaks or gaps along their length but areas showing shorter actin filaments were evident at the tip of stereocilia (Figs. 3b–3d). Tip links connecting the tip of shorter stereocilia to the side of taller ones were found sporadically in the whirler mutants (Fig. 3b) as well as in control hair bundles. Using high resolution TEM we were able to show that the cross-links between the actin filaments in both wildtype and heterozygote stereocilia formed a transverse periodicity across the actin bundle similar to that shown previously for birds [Tilney et al., 1980; Tilney and DeRosier, 1986] (Figs. 4b and 4c). Cross-links between the actin filaments were present in whirler homozygote stereocilia and their alignment also showed a transverse periodicity (Fig. 4e). Most interestingly, sections orthogonal to the actin bundle revealed that the actin filaments were organised in a hexagonal pattern with characteristic 120° angles in both control and whirler homozygotes (Figs. 5c and 5d). Hexagonal actin filament packing has previously been reported for bird stereocilia [Tilney et al., 1983]. Furthermore, actin–tomembrane links were evident in the whirler homozygote stereocilia and they did not differ morphologically from those of the wildtype (Figs. 4a and 4d) or heterozygous animals (data not shown). Comparative TEM analyses of the stereocilia rootlets did not reveal any differences between the whirler homozygotes and their control littermates (Figs. 3a and 3d and data not shown). However, instead of the uniform distinct band of electron dense material at the tips of the stereocilia in controls (Figs. 3a and 3c inset), whirler mutants showed discontinous electron dense patches in their tips (Figs. 3c and 3d).

Quantitative analysis did not reveal a significant difference in the number of actin filaments (+/+: 1174 ± 123, *n* = 11; *wi*/*wi*: 1197 ± 164, *n* = 11; *P* = 0.7) in the wider stereocilia of whirler mice compared to those of the controls (Fig. 2b, see also Figs. 5a and 5b for cross–sectional profiles of the stereocilia). In order to analyse whether increase in whirler stereocilia width corresponds to reduced packing density of the actin filaments, the centre-to-centre distances between the actin filaments were measured and the average number of actin filaments per μm^2^ was calculated (based on actin filament counts within arbitrarily placed squares, see Methods). We found that the lower density of actin filaments in whirler stereocilia compared to control (*wi*/*wi*: 6947 ± 758 per μm^2^, *n* = 76 squares; +/+: 7612 ± 561 per μm2, *n* = 30 squares; *P* < 0.05) corresponded to increased distances between them (*wi*/*wi*: 12.55 ± 1.68 nm, *n* = 337; +/+: 11.74 ± 1.78 nm, *n* = 137; *P* < 0.05). The average nearest-neighbour distances between actin filaments measured within whirler and control stereocilia were slightly smaller than the estimated nearest-neighbour distances calculated for a truly random distribution of filaments within the measured cross-sectional area and counted number of actin filaments [Hsieh et al., 1994] (14.79 and 12.35 nm respectively). This difference between measured and predicted distances can be explained by the existence of imperfections in the patterning within the actin bundle (ie. single filaments missing). The predicted number of actin filaments per stereocilium calculated for an average cross-sectional area of the actin bundle (179,360 nm^2^) within control stereocilia and based on measured distances between filaments was 1235 and did not differ significantly from the actual counts (1174 ± 123 actin filaments per control stereocilium). However, if we use the estimated packing density of actin filaments in wildtype stereocilia to do the same calculation of number of filaments within an average cross-sectional area of the whirler actin bundle (262,256 nm2), then this would predict a total of 1717 filaments, which is noticeably higher than the average number of actin filaments actually counted (1197 ± 164). These results suggest that the actin filament spacing within the whirler mutant stereocilia is greater than in the wildtype and could account for their greater width.

### Stereocilia of Whirler Outer Hair Cells are Fewer in Number and More Widely Spaced

**Fig. 6:**
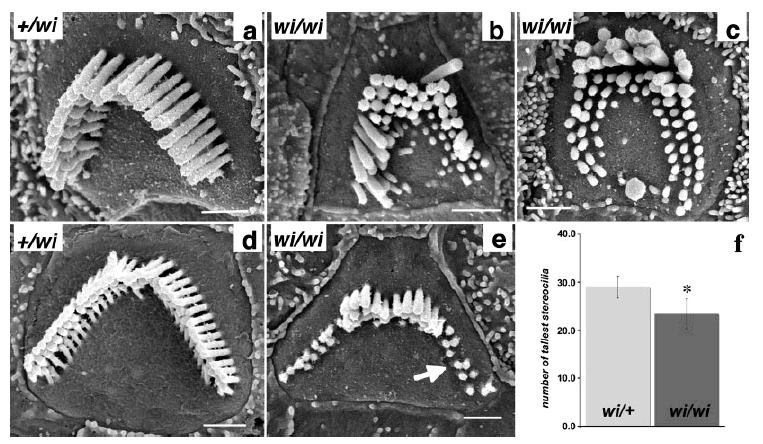
The hair bundles in whirler outer hair cells are irregular. SEM images of outer hair cell stereocilia bundles of +/*wi* (**a**,**d**) and *wi*/*wi* (**b**,**c**,**e**) at P20. An irregular mixture of stereocilia of variable length within a single bundle is often found in the apical turn of the *wi*/*wi* cochlea (b,c). In contrast, in the middle turn the stereocilia appear to be shorter at the ends of the rows and here the number of rows is often reduced to one or two (e, arrow). **f:** A marked reduction in the number of stereocilia of the most lateral row is evident in the whirler outer hair bundle. Standard deviation bars are marked. ^*^ represents a significant difference, *P* < 0.001. Scale bar = 2 μm.

Wildtype and control outer hair cells from the middle turn of the cochlea showed the typical W–shaped arrangement of stereocilia with three rows of graded heights. The stereocilia of whirler homozygotes showed a U–shaped organisation with shorter stereocilia at the end of each rank as previously observed [Holme et al., 2002]. In this study we evaluated the hair bundles of the outer hair cells in greater detail focusing on stereocilia number, length, width and interstereocilia distances within the hair bundle. Hair bundles in the apical region of the cochlea revealed a striking mixture of normal and very short stereocilia within the same row (Figs. 6b and 6c). The distribution of stereocilia of different lengths within the bundle was irregular. In contrast, in the middle and basal cochlear turns the stereocilia lengths decreased with increasing distance from the centre of the hair bundle as previously described [Holme et al., 2002], and there were often only one or two rows of stereocilia remaining towards the ends of the rows instead of the usual three rows (Fig. 6e). Both W or U–shaped stereocilia organisations were observed. In addition, analysis of the hair bundles in the middle turn revealed a significant reduction in the number of the tallest stereocilia in whirler homozygotes (23.0 stereocilia per bundle, *n* = 20; *P* < 0.001) compared with the number in controls (29.0 stereocilia per bundle, *n* = 33) (Fig. 6f).

**Fig. 7:**
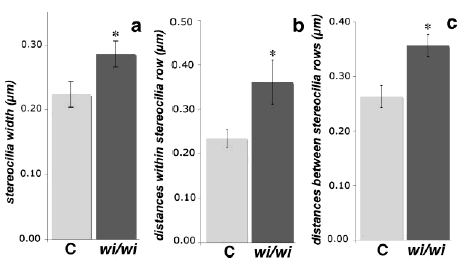
Outer hair cell stereocilia of whirler mutants are wider and further apart than in controls. Statistical analyses of outer hair cell stereocilia reveal not only significant increases in stereocilia width (*P* < 0.001) (**a**) but also in the distances between the stereocilia within a row (*P* < 0.001) (**b**) and between rows (*P* < 0.001) (**c**). Standard deviation bars are marked. ^*^ represents a significant difference, *P* < 0.001.

TEM analysis showed a significant increase in outer hair cell stereocilia diameter in whirler homozygotes (+/+: 0.22 ± 0.02 μm, *n* = 51; *wi*/*wi*: 0.29 ± 0.02 μm, *n* = 51; *P* < 0.001) (Fig. 7a). In addition, the centre-to-centre spacings of the stereocilia within rows (+/+: 0.23 ± 0.02 μm, *n* = 51; *wi*/*wi*: 0.36 ± 0.02 μm, *n* = 51; *P* < 0.001) and between rows (+/+: 0.26 ± 0.02 μm, *n* = 33; *wi*/*wi*: 0.36 ± 0.04 μm, *n* = 51; *P* < 0.001) were significantly greater in the whirler homozygotes than in the control (Figs. 7b and 7c). SEM images revealed the presence of tip links and horizontal top connectors between the stereocilia in both control and whirler homozygotes (Figs. 8a–8c). TEM images of control outer hair cells showed horizontal connectors linking adjacent stereocilia (about 0.1 μm wide) with a distinct dark line in the middle [Furness and Hackney, 1985; Goodyear et al., 2005] (Fig. 7d). These links in the whirler outer hair cells appeared more stretched (about half the width of the control) and lacked the distinct dark middle line (Fig. 7e). However, quantitative analysis was not possible as only a relatively small number of high quality images were obtained. Membrane–to–actin links were evident in outer hair cell stereocilia of both control and whirler mice (data not shown).

**Fig. 08:**
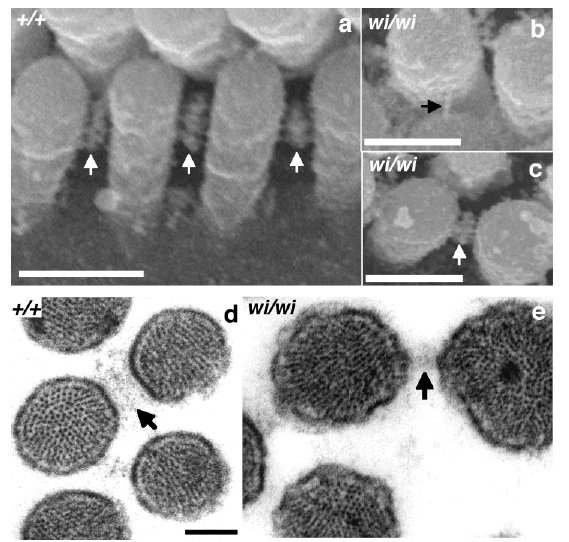
Outer hair cell stereocilia of whirler mutants have stretched horizontal connectors. SEM and TEM images reveal the presence of tip links and horizontal top connectors in wildtype and whirler homozygotes. SEM images showing a lateral view of wildtype, +/+, stereocilia interlinked by horizontal top connectors (**a**, arrows) and apical views of *wi*/*wi* stereocilia showing the presence of tip links (**b**, arrow) and horizontal top connectors (**c**, arrow). High resolution TEM images showing that the extracellular links (most likely the horizontal connectors, arrows) linking adjoining stereocilia in *wi*/*wi* (**e**) are morphologically different (lacking a distinct dark band in the middle and appearing more stretched) from those of the control (**d**). Scale bars: a-c = 200 nm; d,e = 100 nm.

## DISCUSSION

This study further characterises the effects that a lack of the novel PDZ protein whirlin has on stereocilia organisation and dimensions in the inner and outer hair bundles. The cellular arrangement of the whirler mutant organ of Corti is normal, showing three rows of outer and one row of inner hair cells separated by apparently normal supporting cells [Holme et al., 2002]. Many features of hair bundle structure are normal in whirler homozygotes: the whirler stereocilia grow and are arranged in rows of graded heights (staircase-like), they are oriented correctly with the tallest stereocilia towards the lateral edge of the hair cell, they show tip links and horizontal connectors between adjacent stereocilia, the actin core is present forming a paracrystalline array of hexagonally packed continuous actin filaments and links between the actin core and the cell membrane appear normal as in wildtype. However, we show a number of clear defects in mature whirler hair bundles. The inner hair cell stereocilia are shorter and wider than in controls and the increase in diameter is not accompanied by an increase in the number of actin filaments but is due to a reduction in the packing density of the actin core. Furthermore, the tip-complex is reduced to patches of electron dense material in the stereocilia of whirler homozygotes. We found that the number of outer hair cell stereocilia in the tallest row was reduced and that the centre-to-centre spacing between the stereocilia was greater in the absence of whirlin. These abnormalities are summarised in Table I.

**TABLE I:** Summary of the Abnormal Features of Whirler Inner and Outer Hair Cell Hair Bundles

Inner hair cells of *wi/wi*	Outer hair cells of*wi/wi*
Stereocilia shorter and wider than those of control8	Stereocilia wider than those of control
Looser packing density of actin filaments in stereocilia core than control	Not measured
Electron density in stereocilia tips reduced compared with control	Not assessed
Shape of hair bundle more oval than in controls	Shape of hair bundle more U-shaped than V-shaped
Extra rows of stereocilia (4–5 instead of 3)	Reduced number of stereocilia rows (1 or 2 rows in places)
Increased stereocilia packing density within each row (but not between rows)	Reduced stereocilia packing density within each row and between rows

## Lack of Whirlin Affects Actin Filament Elongation and Packing

The actin filaments of the core of mammalian stereocilia undergo a process of treadmilling where polymerisation of actin filaments at the stereocilium tip is balanced by filament depolymerisation at its base so that stereocilium length is maintained in a dynamic steadystate. Experiments utilising cytochalasin D have shown that the processes of actin polymerisation and depolymerisation in the stereocilia are independent and that inhibition of actin polymerisation does not affect the rate of depolymerisation [Rzadzinska et al., 2004]. Evidently, tight regulation of actin filament assembly at the tip and its disassembly at the base is needed for overall elongation and stereocilia growth to occur. Our previous studies have shown that inner hair cell stereocilia of mice lacking whirlin are already shorter by embryonic day 18.5 (E18.5) and decrease in length during early postnatal development and eventually the hair cells degenerate around P60 [Holme et al., 2002]. Developmental expression patterns for whirlin have shown that it is present transiently in stereocilia tips and that the time window is different for the rows of stereocilia of differing heights, but ranges from around birth to postnatal day 12 [Kikkawa et al., 2005]. This suggests that whirlin is not essential for actin nucleation or initial actin filament assembly (as this occurs prior to E18.5) but that it is likely to play a role in stereocilia elongation. It is tempting to speculate that whirlin may promote continued actin filament elongation by inhibiting capping proteins during postnatal stereocilium development and maturation. Once the full stereocilium length has been reached capping proteins may then cap the barbed-ends preventing further elongation while presumably allowing slow turnover. This hypothesis is consistent with the reported fade out of whirlin at the tip of the stereocilia by P12 in inner hair cells [Kikkawa et al., 2005]. In the absence of whirlin, the barbed ends of the actin filaments may be capped prematurely thus preventing further actin filament and consequently stereocilium elongation. In addition, whirlin as a component of the tip-complex may play a role in anchoring and positioning the actin filaments at the stereocilium tip as discontinuity of the tip density corresponds with shorter actin filaments in whirler homozygotes. The electron dense material, referred to as the tipcomplex, has previously been reported not only at the tip of stereocilia [DeRosier and Tilney, 2000] but also at the tip of filopodia and it has been implicated in the regulation of actin filament elongation [Svitkina et al., 2003]. A diminished tip-complex could affect actin turnover at the barbed-end by slowing down the polymerisation rate and thus causing net actin filament shrinkage followed by stereocilium shortening as observed in the whirler homozygote.

Very similar changes in stereocilia length, but accompanied by a reduction in stereocilia diameter, have been described for the jerker mouse mutant, which lacks the actin crosslinker espin [Anniko et al., 1989; Sjostrom and Anniko, 1992; Zheng et al., 2000; Rzadzinska et al., 2005]. Similarly, *Drosophila* bristles lacking one or more actin crosslinkers shorten progressively until they disappear due to an increase in the actin depolymerization rate [Guild et al., 2005]. The shortening of mutant bristles was accompanied by a decreasing diameter presumably due to faster depolymerization in the most peripheral filaments as they have fewer cross-links. The inner hair cell stereocilia of whirler homozygotes also shorten during postnatal maturation but only until they reach a certain length. Interestingly, the diameter of the whirler homozygote stereocilia increases while the number of actin filaments is not significantly different from that of the control. Shortening of the whirler stereocilia could therefore also be a consequence of the increased distances between the actin filaments, which may produce a less stable actin core, and promote faster depolymerization. However, the whirler stereocilia did not show any obvious reduction in actin filament cross-links, which may explain why they do not disappear completely. It is also tempting to speculate that whirlin may influence the bundling of actin filaments at the stereocilium tip [Rzadzinska et al., 2005].

## The Whirler Phenotype is Different From That of Shaker2

Recent studies suggest that whirlin interacts with myosin XVa *in vitro* and that myosin XVa is critical for the transport of whirlin to the tip of the stereocilia [Belyantseva et al., 2005; Delprat et al., 2005]. The shaker2 phenotype has therefore been suggested to be a result of a lack of whirlin during stereocilia development. However, our analyses of the organisation and ultrastructure of the whirler homozygote have revealed distinct differences compared with the published data on the shaker2 mutant. Mice homozygous for the whirler allele showed almost normal stereocilia bundles at P4 whereas those of the shaker2 homozygote were very short at P9 [Holme et al., 2002; Stepanyan et al., 2006]. Furthermore, significant deviations from the normal 3 rows of stereocilia were observed in the whirler mutant (with four to five rows in inner hair cells and frequent reduction to two rows in outer hair cells) that has not been reported for the shaker2 mice. Also the characteristic long actin bundles (cytocauds) [Probst et al., 1998; Beyer et al., 2000; Kanzaki et al., 2002] found to extend from the base of the shaker2 hair bundle toward the base of the hair cell [Probst et al., 1998] were not found in the auditory epithelium of whirler homozygotes. In addition, we have shown that patches of electron dense material are present at the tip of whirler stereocilia while in the shaker2 stereocilia such electron density is completely lacking. Although these differences do not preclude an interaction between whirlin and myosin XVa, they suggest that these proteins also play separate roles in stereocilia elongation, width determination and maintenance which are yet to be uncovered. Furthermore, the fact that the tip density is completely lacking in shaker2 but only reduced in whirler suggests that myosin XVa may act as a motor not only for whirlin but possibly also for other tip-complex proteins. However, recent measurements of single hair cell responses in shaker2 and whirler mutants have demonstrated transduction currents with many normal features in immature apical outer hair cells of both mutants, indicating that neither myosin XVa nor whirlin are essential for the formation and function of the mechanotransduction complex [Stepanyan et al., 2006], which is also supported by the presence of tip links in the whirler homozygotes as shown in this study.

## Lack of Whirlin Affects the Stereocilia of the Inner and Outer Hair Cells in Different Ways

Our results reveal that the whirler mutation has different effects on the organisation and morphology of the stereocilia bundles of inner and outer hair cells (Table I). The inner hair cell stereocilia of whirler mutants shorten during early postnatal development [Holme et al., 2002] and reach a length of well under half that of the control by P20, and this is accompanied by an increase in diameter. The number of stereocilia within each inner hair cell bundle was greater in whirler mutants than in the controls. In contrast, the stereocilia of whirler outer hair cells did not shorten uniformly but formed a bundle with the tallest stereocilia nearest the centre with fewer and shorter stereocilia further towards the ends of the rows, or consisted of irregularly positioned stretches of long and short stereocilia with an overall reduction in number. Despite significant differences in the general bundle morphology compared with inner hair cell bundles, the stereocilia of whirler outer hair cells were also wider than the controls. These differences indicate that whirlin plays different roles in the development and maturation of the outer and inner hair cells and that the overall effect of whirlin is likely to depend on its specific developmental expression pattern in these cells and on the presence of other molecular players. Recently, the erthryocyte protein p55 (a membrane-associated guanylate kinase, MAGUK, protein) and a second erythrocyte-expressed protein 4.1R were shown to interact with whirlin [Mburu et al., 2006]. Both of these proteins localize to the stereocilia in the outer hair cell but not in the inner hair cell and both show a very specific developmental distribution. Whirlin has recently been reported to interact with USH2A [Adato et al., 2005], a component of the ankle links. Interestingly, despite the major remodelling of the whirler hair bundles and shrinkage of the stereocilia, the links between the actin core and the plasma membrane as well as the interstereocilial links are present, and it is only at the TEM level that the horizontal connectors appeared morphologically different from the control. Future immuno-gold localisation and protein interaction studies will be important to unravel the function of whirlin in actin filament elongation, linkage to the membrane and in interstereocilia links.
